# Presence of Tuft Cells Expressing Hematopoietic Prostaglandin D Synthase in Acinar-to-Ductal Metaplasia in Human Obstructive Pancreatitis

**DOI:** 10.3390/cimb48060585

**Published:** 2026-06-02

**Authors:** Kenta Hosomi, Mitsuaki Ishida, Kensuke Nakanishi, Kohei Taniguchi, Jun Arima, Atsushi Tomioka, Mitsuhiro Asakuma, Sang-Woong Lee, Ko Fujimori, Yoshinobu Hirose

**Affiliations:** 1 Department of Pathobiochemistry, Faculty of Pharmacy, Osaka Medical and Pharmaceutical University, 4-20-1, Nasahara, Takatsuki City 569-1094, Osaka, Japan; ompu72123058@s.ompu.ac.jp (K.H.); ko.fujimori@ompu.ac.jp (K.F.); 2 Department of Pathology, Faculty of Medicine, Osaka Medical and Pharmaceutical University, 2-7, Daigaku-Machi, Takatsuki City 569-8686, Osaka, Japan; kensuke.nakanishi@ompu.ac.jp (K.N.); yoshinobu.hirose@ompu.ac.jp (Y.H.); 3 Division of Pathology, Osaka Medical and Pharmaceutical University Hospital, 2-7, Daigaku-Machi, Takatsuki City 569-8686, Osaka, Japan; 4 Department of General and Gastroenterological Surgery, Faculty of Medicine, Osaka Medical and Pharmaceutical University, 2-7, Daigaku-Machi, Takatsuki City 569-8686, Osaka, Japan; jun.arima@ompu.ac.jp (J.A.); atsushi.tomioka@ompu.ac.jp (A.T.); mitsuhiro.asakuma@ompu.ac.jp (M.A.); sang-woong.lee@ompu.ac.jp (S.-W.L.); 5 Center for Medical Research & Development, Division of Translational Research, Faculty of Medicine, Osaka Medical and Pharmaceutical University, 2-7, Daigaku-Machi, Takatsuki City 569-8686, Osaka, Japan; kohei.taniguchi@ompu.ac.jp

**Keywords:** pancreas, tuft cell, acinar-to-ductal metaplasia, obstructive pancreatitis, prostaglandin D_2_

## Abstract

Acinar-to-ductal metaplasia (ADM)—a process involving the dedifferentiation or transdifferentiation of pancreatic acinar cells—is recognized as an initial event in pancreatic tumorigenesis. Studies in mouse models have revealed that tuft cells, which are chemosensory epithelial cells, appear in ADM following tissue injury, and tuft cell-produced prostaglandin (PG) D_2_ may suppress inflammation and tumorigenesis. However, the presence and role of tuft cells in the human pancreas remain unclear. Therefore, in this study, we investigated the presence of tuft cells and PGD_2_ production in human ADM. We analyzed ADM lesions from consecutive patients undergoing surgical resection for pancreatic tumors using dual immunohistochemical staining for POU domain class 2 transcription factor 3 (POU2F3) and hematopoietic PGD synthase (H-PGDS). All 29 patients (13 men and 16 women) with diagnoses including pancreatic ductal adenocarcinoma and intraductal papillary mucinous neoplasms exhibited ADM in regions of obstructive pancreatitis. Immunohistochemical analysis showed that 67.3% of ADM lesions contained POU2F3- and/or H-PGDS-positive cells. Among these, 85.5% of POU2F3-positive cells co-expressed H-PGDS, and 76.2% of H-PGDS-positive cells were POU2F3-positive. These findings indicate that tuft cells present in human ADM produce PGD_2_, suggesting a role in tissue repair. Tuft cells may represent a potential therapeutic target in pancreatitis, warranting further investigation into their functional role in ADM.

## 1. Introduction

Pancreatic ductal adenocarcinoma (PDAC) is a leading cause of cancer-related death worldwide, and the molecular mechanisms underlying its development have been extensively studied [1]. Recently, acinar-to-ductal metaplasia (ADM) has attracted attention as an initiating event in pancreatic tumorigenesis [2,3]. ADM is a process in which pancreatic acinar cells undergo dedifferentiation or transdifferentiation, acquiring features of both embryonic progenitor-like and ductal cells [2,3,4,5]. Histologically, ADM is characterized by expansion of the acinar lumina, which are lined with flattened, zymogen-depleted cells resembling pancreatic ductules, representing a condition previously termed a tubular complex [6,7].

ADM can be induced by various factors, including inflammation and other persistent environmental stresses [2,4,8]. Consequently, ADM is considered a mechanism by which acinar cells are regenerated, thereby supporting pancreatic tissue repair following injury [2,3,4,5]. ADM is frequently observed in acute or chronic pancreatitis [3,4,5,7] and in pancreatic tissue adjacent to pancreatic tumors, particularly in obstructive pancreatitis [7]. The research employing *Kras*^G12D^ transgenic mouse models suggests that ADM may progress to pancreatic intraepithelial neoplasia (PanIN), a potential precursor to PDAC [9]. However, the exact pathway from ADM to PanIN in the human pancreas remains unclear.

Tuft cells are chemosensory epithelial cells predominantly found in the digestive and respiratory tracts [10,11]. POU domain class 2 transcription factor 3 (POU2F3) is a key regulator of tuft-cell differentiation [12]. Tuft cells are involved in antibacterial defense, initiation of immune responses, and promotion of tissue repair [13,14], and several studies have revealed their pivotal role in pancreatitis [13,14,15]. Recent mouse-model study has shown that acinar cells can transdifferentiate into tuft cells within ADM regions of mice in response to tissue injury [16]. Moreover, DelGiorno et al. demonstrated that tuft cells inhibit the development and acceleration of PanIN to PDAC in the *Kras*^G12D^ transgenic mouse model [9].

Prostaglandins (PGs) are lipid mediators that play important roles in regulating various physiological activities, including inflammation, vasodilatation, and smooth-muscle contraction or dilatation [17]. PGs are synthesized from arachidonic acid via the action of cyclooxygenases (COXs) and specific PG synthases [18]. PGD_2_, in particular, is synthesized from PGH_2_ by PGD synthases (PGDSs), which exists in two distinct forms: hematopoietic PGDS (H-PGDS) and lipocalin-type PGDS (L-PGDS) [19]. H-PGDS is primarily involved in the production of PGD_2_ in immune cells such as macrophages, mast cells, and a subset of T lymphocytes (Th2), whereas L-PGDS is mainly expressed in the brain and heart [20,21].

Previous studies have reported that PGD_2_ regulates inflammation in mice [17], and 15-deoxyΔ^12,14^-PGJ_2_—a metabolite of PGD_2_—attenuates the severity of acute pancreatitis [22]. Notably, PGD_2_ released from tuft cells during ADM can suppress the development and acceleration of PanIN to PDAC in *Kras*-induced pancreatic tumorigenesis mouse models [9]. In human pancreas, presence of tuft cells in chronic pancreatitis tissues using immunohistochemical staining for phosphorylated-epidermal growth factor receptor, one of tuft cell markers, and in situ RNA sequence, has been reported; however, no detailed information on the location of tuft cells were available [9,23]. Our recent study demonstrated the presence of tuft cells in human ADM lesions [24]. However, the role of PGD_2_ in ADM of the human pancreas remains unclear. Therefore, this study aimed to analyze the expression of H-PGDS in ADM of human pancreatic tissues and to explore the potential role of tuft cells in human pancreatic injury and tumorigenesis.

## 2. Materials and Methods

### 2.1. Patient Selection

We selected consecutive patients with pancreatic tumors who underwent surgical resection at the Department of General and Gastroenterological Surgery of Osaka Medical and Pharmaceutical University Hospital between January 2022 and December 2023. Patients who had received neoadjuvant chemotherapy and/or radiation therapy were excluded. Initially, the present cohort included 76 pancreatic tumor patients who received surgical resection (40 males and 36 females; median age was 74 years, range 47–86 years). Histopathological diagnoses included intraductal papillary mucinous neoplasm (IPMN) with low-grade dysplasia in 12 patients, IPMN with high-grade or associated invasive carcinoma in 16 patients, PDAC in 46 patients, and one patient each of solid pseudopapillary neoplasm and neuroendocrine tumor coexisting with IPMN with low-grade dysplasia (pStages 0, 1, 2, 3, and 4 in 15, 18, 2, 39, and 2 patients, respectively). Forty-one patients who received neoadjuvant therapy and 6 patients with insufficient pancreas tissues for immunohistochemical analysis were excluded.

The present cohort was fundamentally the same as that used in our previous study [24], which examined the presence of tuft cells in ADM and PanIN. However, the present analysis focuses on the expression of H-PGDS in tuft cells in ADM and does not overlap with the previously reported findings [24].

This retrospective single-institution study was conducted in accordance with the principles of the Declaration of Helsinki, and the study protocol was approved by the Institutional Review Board of Osaka Medical and Pharmaceutical University (Approval No. 2023-198). All data were anonymized. The Institutional Review Board waived the requirement for informed consent because of the retrospective study design and the use of anonymized medical records and archived samples; moreover, the present study did not include minors. Study information, including inclusion criteria and the opportunity to opt out, was made available on the institutional website (https://www.ompu.ac.jp/u-deps/path/img/file34.pdf, accessed on 12 May 2026).

### 2.2. Histopathological Analysis

Surgically resected specimens were fixed in 10% neutral buffered formalin, sectioned, and stained with hematoxylin and eosin. All slides were histopathologically examined for the presence of ADM in regions of obstructive pancreatitis. ADM was defined as the widening of the acinar lumina lined by flattened cells without zymogen granules, in accordance with previous studies [6,7]. Histopathological evaluation was performed independently by three researchers.

### 2.3. Immunohistochemical Analysis

Immunohistochemical staining was performed using an autostainer (Leica Bond-MAX; Leica Biosystems GmbH, Nußloch, Germany) according to the manufacturer’s instructions. The BOND Polymer Refine Detection Kit (DS9800; Leica Biosystems GmbH) and the BOND Polymer Refine Red Detection Kit (DS9390; Leica Biosystems GmbH) were used for dual immunohistochemical staining. A rabbit monoclonal antibody against POU2F3 (E5N2D; Cell Signaling Technology, Danvers, MA, USA; diluted 1:200) and a rabbit polyclonal antibody against H-PGDS (this antibody was the same as that used in a previous study [25,26]; diluted 1:4000) were used. As positive controls, squamous cells of the skin were used for POU2F3 [26] and placental trophoblasts were used for H-PGDS [27]. Negative controls were prepared without the primary antibodies. Nuclear staining was considered positive for POU2F3 [27], whereas both nuclear and cytoplasmic staining were considered positive for H-PGDS [28].

Furthermore, one or two representative slides from all surgically resected specimens containing ADM were selected for immunostaining. Finally, immunohistochemical features were independently evaluated by two researchers.

## 3. Results

### 3.1. Patient Characteristics

This study included 29 patients (13 men and 16 women) with a median age of 74 years (range: 51–84 years) at the time of surgery. Tumors were located in the pancreatic head in 11 patients (37.9%) and body and tail in 18 patients (62.1%). Histopathological diagnoses included IPMN with low-grade dysplasia in 10 patients, IPMN with high-grade dysplasia or associated invasive carcinoma in 10 patients, PDAC in seven patients, and one patient each of solid pseudopapillary neoplasm and neuroendocrine tumor coexisting with IPMN with low-grade dysplasia (pStages 0, 1, 2, and 3 in 13, 6, 2, and 7 patients, respectively).

### 3.2. ADM in Obstructive Pancreatitis

**Figure 1:**
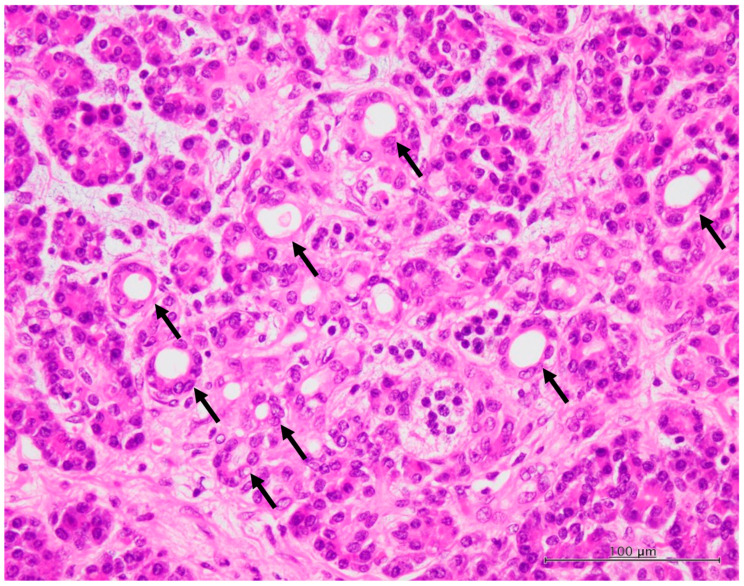
Histopathological features of acinar-to-ductal metaplasia in obstructive pancreatitis. Acinar-to-ductal metaplasia is characterized by the presence of ductal-like lumina lined by flattened cells without zymogen granules in the pancreatic acini (arrows) (hematoxylin and eosin, ×400).

We investigated the presence of ADM in obstructive pancreatitis lesions across all 29 patients. ADM was identified in the surrounding pancreatic tissues of all patients. Typical histopathological features of ADM are shown in Figure 1. ADM is histopathologically characterized by the widening of the acinar lumina and presence of ductal-like lumina in the pancreatic acini. These cells composed of the lumina were flattened in shape and lacked visible zymogen granules (Figure 1).

### 3.3. Tuft Cells Expressed H-PGDS

**Figure 2:**
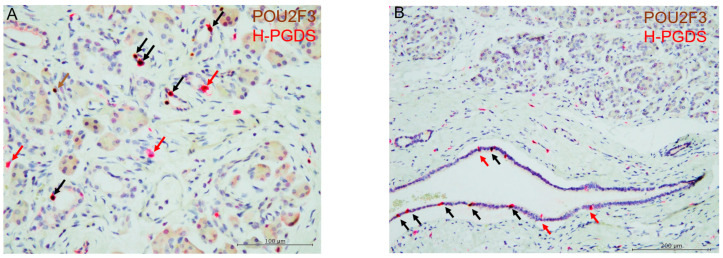
Dual immunohistochemical staining. (**A**) POU2F3 (brown)-positive tuft cells also express H-PGDS (red). POU2F3 and H-PGDS double-positive cells are black in nuclei and red in cytoplasm (black arrows). H-PGDS single-positive cells are also present (red arrows) (×400). (**B**) POU2F3-positive tuft cells are absent in the normal acini. POU2F3 and H-PGDS double-positive cells are scattered in the pancreatic duct (black arrows), and H-PGDS-positive cells are also noted in the pancreatic duct (red arrows) (×200).

Dual immunohistochemical staining showed expression of H-PGDS in tuft cells (Figure 2A). Tuft cells were identified by POU2F3 expression in the nucleus (brown), while H-PGDS was localized in both the nucleus and the cytoplasm (red). Cells positive for POU2F3 and/or H-PGDS were observed in 67.3% of ADM lesions (705 of 1046 lesions), with the remaining 32.7% lacking both POU2F3 and H-PGDS expression. Dual immunohistochemical staining for ADM in human obstructive pancreatitis revealed the following cell counts: among POU2F3-positive tuft cells, 85.5% also expressed H-PGDS (1796 of 2100 cells) and 14.5% (304 of 2100 cells) were H-PGDS-negative, while 76.2% of H-PGDS-positive cells were also POU2F3-positive (1796 of 2356 cells) and 23.8% (560 of 2356 cells) were POU2F3-negative.

H-PGDS, hematopoietic prostaglandin D synthase; POU2F3, POU domain class 2 transcription factor 3.

In normal pancreatic tissues, no POU2F3-positive tuft cells were detected in the pancreatic acini. A small number of POU2F3-positive tuft cells were observed in the normal pancreatic ducts, most of which also expressed H-PGDS (Figure 2B).

## 4. Discussion

The present study clearly demonstrated that 85.5% of tuft cells present in human ADM expressed H-PGDS, suggesting that these cells produced PGD_2_. To our knowledge, this is the first report of H-PGDS expression in tuft cells in ADM of the human pancreas, indicating that tuft cells may play important roles in tissue repair following acinar cell injury.

PGD_2_ is produced by H-PGDS or L-PGDS downstream of COX1 or COX2 [17,18] and plays multiple roles in inflammation and homeostasis via D-prostanoid 1 (DP1) or chemoattractant receptor-homologous molecule expressed on Th2 cells (CRTH2) receptors [17]. For example, DP1 promotes Th2 differentiation during allergic inflammation [29], while CRTH2 is expressed on Th2 lymphocytes, eosinophils, basophils, group 2 innate lymphoid cells, and small intestinal epithelial cells, thereby contributing to inflammatory responses [30,31,32]. Accordingly, PGD_2_ may exert both pro- or anti-inflammatory effects depending on the environment [33].

PGD_2_ is also implicated in tumorigenesis. It can recruit macrophages, enhance antitumor chemokine and cytokine production, and suppress pathways involved in expression of survival genes and metalloproteinases, epithelial–mesenchymal transition transcription, angiogenesis, and apoptosis of tumor cells, resulting in antitumor effects [34]. Consequently, high expression of PGD_2_ is associated with improved prognosis in various carcinomas, including gastric, colorectal, pancreatic, and breast cancer, with macrophages identified as a primary source of PGD_2_ [34].

Tuft cells are chemosensory epithelial cells distinguished by a tuft of long and thick microvilli on their apical surface [10,11,14,35]. Recognized as sensors for detection of various chemical signals, they respond by secreting different physiologically active substances [10,11,14,35]. In the intestine, tuft cells produce interleukin-25 (IL-25) in response to parasitic infections, triggering type 2 inflammation and tissue remodeling [14,35]. In the nasal epithelium, tuft cells generate acetylcholine, which combats bacteria through the release of antimicrobial peptides, inflammation, and the sneezing reflex [35]. In this study, we confirmed the presence of tuft cells in the ducts of the human pancreas, by not in the acini, and this finding aligns with previous reports identifying tuft cells using choline acetyltransferase [36]. Additionally, this study revealed for the first time that tuft cells in normal human pancreatic ducts express H-PGDS. While tuft cells were previously known to express COX1 or COX2 [10,11,14,34], our findings suggest that they may play a role in managing inflammation and/or tissue repair in the normal pancreatic duct via production of various physiologically active substances, including PGD_2_ [35].

Our previous study, which examined the presence and roles of POU2F3-positive tuft cells in human pancreatic tumorigenesis, showed that POU2F3-positive tuft cells were present in 46.4% of ADM lesions, were higher in low-grade PanIN compared to in high-grade PanIN in the human pancreas, and were absent in normal pancreatic acini [24]. However, whether these tuft cells produce PGD_2_ in human ADM and their actual function in ADM remains unclear. The present study clearly demonstrated for the first time that 67.3% of ADM lesions contained POU2F3- and/or H-PGDS-positive cells, and that 85.5% of tuft cells in human ADM lesions expressed H-PGDS. Given that H-PGDS immunohistochemical staining is a reliable indicator of PGD_2_ production [9,20,26], our findings suggest that tuft cells present in human ADM are one of the main sources of PGD_2_. This may indicate an important role for PGD_2_ in tissue repair in the human pancreatic acini, consistent with the findings from mouse-model study [9].

This study had some limitations. First, this study included small numbers of pancreatic tissues from 29 patients. Thus, a selection bias cannot be ruled out, although 1046 ADM lesions were analyzed. Second, the functional role of PGD_2_ in human ADM remains unclear and the distributions and functions of PGD_2_ receptors, DP1 and CRTH2, in human ADM have been unresolved. Although PGD_2_ produced by tuft cells may contribute to tissue repair, its molecular mechanisms in human ADM lesions have not been elucidated. Further studies are therefore needed, particularly given the potential of tuft cells as therapeutic targets in pancreatitis [37]. Third, the distributions of POU2F3 and H-PGDS in human ADM lesions were not completely concordant. In this study, 76.2% of H-PGDS-positive cells were also POU2F3-positive, while the remaining 23.8% of H-PGDS-positive cells were POU2F3-negative. This may be because some slices of dual immunostaining only included the cytoplasm of tuft cells and not the nuclei (POU2F3 expression is limited to nuclei [25,27]). However, the identity of most of these H-PGDS-positive/POU2F3-negative cells remained unclear, and further studies are needed to clarify the cellular sources of PGD_2_ in human ADM lesions. Finally, POU2F3 was used as the human tuft-cell marker in this study. Other tuft-cell markers, such as doublecortin-like kinase 1 (DLCK1), were not assessed. However, DCLK-1 is commonly used in mouse models and is not specific for human tuft cells [14], whereas POU2F3 is considered a master regulator of tuft-cell identity [14]. Accordingly, POU2F3 was selected as the human tuft-cell marker in the present study.

## 5. Conclusions

This study demonstrates that tuft cells in human ADM produce PGD_2_, indicating that they play an important role in tissue repair. These findings highlight tuft cells as a potential therapeutic target in pancreatitis. Therefore, further studies are needed to clarify the function of tuft cells in ADM.

## Data Availability

The original contributions presented in this study are included in the article. Further inquiries can be directed to the corresponding author.

## Abbreviations

The following abbreviations are used in this manuscript:

ADM	acinar-to-ductal metaplasia
COX	cyclooxygenase
CRTH2	chemoattractant receptor-homologous molecule expressed on Th2 cells
DLCK1	doublecortin-like kinase 1
DP1	D-prostanoid 1
H-PGDS	hematopoietic prostaglandin D synthase
IPMN	intraductal papillary mucinous neoplasm
L-PGDS	lipocalin-type prostaglandin D synthase
PanIN	pancreatic intraepithelial neoplasia
PDAC	pancreatic ductal adenocarcinoma
PGD_2_	prostaglandin D_2_
POU2F3	POU domain class 2 transcription factor 3
